# Metabolic Mechanism of Sulfadimethoxine Biodegradation by *Chlorella* sp. L38 and *Phaeodactylum tricornutum* MASCC-0025

**DOI:** 10.3389/fmicb.2022.840562

**Published:** 2022-03-18

**Authors:** Bing Li, Di Wu, Yan Li, Yan Shi, Chenlin Wang, Jiasi Sun, Chunfeng Song

**Affiliations:** ^1^Tianjin Academy of Agricultural Sciences, The Institute of Agriculture Resources and Environmental Sciences, Tianjin, China; ^2^Tianjin Key Laboratory of Indoor Air Environmental Quality Control, School of Environmental Science and Engineering, Tianjin University, Tianjin, China

**Keywords:** sulfadimethoxine, *Chlorella* sp., *Phaeodactylum tricornutum*, microalgae, NaCl, biodegradation

## Abstract

Antibiotic resistance is one of the most important environmental challenges. Microalgae has been considered as a promising green media for environmental purification. In this work, sulfadimethoxine (SDM) biodegradation potential of *Chlorella* sp. L38 and *Phaeodactylum tricornutum* MASCC-0025 is investigated. Experimental results indicated that the tested freshwater and marine microalgae strains presented stress response to SDM addition. For *Chlorella* sp. L38, it has a good adaptability to SDM condition *via* antioxidant enzyme secretion (SOD, MDA, and CAT up to 23.27 U/mg, 21.99 μmol/g, and 0.31 nmol/min/mg) with removal rate around 88%. *P. tricornutum* MASCC-0025 exhibited 100% removal of 0.5 mg/L SDM. With increasing salinity (adding a certain amount of NaCl) of cultivation media, the removal rate of SDM by microalgae increased. Although its adaptive process was slower than *Chlorella* sp. L38, the salinity advantage would facilitate enzyme accumulation. It indicated that microalgae could be used to remove SDM from freshwater and marine environment *via* suitable microalgae strain screening.

## Introduction

Sulfonamides are one of the most frequently used antibiotics for therapeutic purposes and are also used as feed additives in certain intensive farming operations (Peiris et al., 2017). Due to the continuous consumption, sulfonamides, such as sulfadimethoxine (SDM), sulfamethoxazole (SMX), and sulfamethazine (SMZ), have been widely found in wastewater, freshwater, and groundwater (Mulla et al., 2018, 2021). In the last decades, antibiotics utilization in veterinary and human medicine was widespread (0.1–0.2 million tons per annum of antibiotics have been utilized all around the world), which increased the risk of environmental contamination (Homem and Santos, 2011; Kumar and Pal, 2018). For example, being the largest country of antibiotics consumption and production in the world, China consumed 92,700 tons of antibiotics in which 48% are consumed by humans and the remaining 52% are by animals (Zhang et al., 2015; Qiao et al., 2018).

Associated with extensive utilization, they pose potential hazards both environmentally and health wise since they are not easily biodegradable and can cause numerous ecological impacts (e.g., promoting the growth of antibiotic-resistant genes and antibiotic-resistant bacteria) (Shao et al., 2005; Zhou et al., 2009). Until now, the dominant antibiotics removal technologies include chemical oxidation, adsorption, membrane and biodegradation, etc. (Ahmed et al., 2015; Yu et al., 2016; Anjali and Shanthakumar, 2019; Bilal et al., 2019). The operation and maintenance cost of chemical oxidation technology is high. Adsorption method and membrane separation method do not realize the harmlessness of pollutants, and there is a lack of reasonable disposal technology for the separated antibiotics. The growth and metabolism of microorganisms will be affected by antibiotics, resulting in the low treatment efficiency of antibiotics by traditional sewage treatment technology (Ali et al., 2018; Sutherland and Ralph, 2019). In addition, there is also a risk of secondary pollution due to the substantial dosage requirements on sorbents, solvent, flocculants, and other agents (Li et al., 2019; Cheng et al., 2020; Zhang and Li, 2020).

As a green and cost-effective alternative of conventional antibiotics removal techniques, microalgae-based processes have attracted more and more attention in the recent years (Cheng et al., 2017; Leng et al., 2020). Compared with the conventional chemical and physical technologies, microalgae-based processes have presented the advantage of lower capital and operational costs, natural disinfection, and high efficiency (Craggs et al., 2012; Cheng et al., 2017). Meanwhile, application of microalgae for bioremediation purpose provides an opportunity to reuse the contaminants as nutrition *via* photosynthesis process, and produce potential value-added biomass (namely polysaccharides, protein, pigment, and lipid) (Sutherland et al., 2018; Song et al., 2020). From the existing results, some microalgae strains have the capacity to resist to antibiotics in the natural environment and can remove them by various mechanisms (such as biosorption, bioaccumulation, and intracellular/extracellular biodegradation) (Nagarajan et al., 2019; Song et al., 2019, 2020).

It should be noted that the antibiotics biodegradation mechanism would be different for different microalgae species. The objective of this work is to investigate the biodegradation of SDM by two different microalgae, derived from freshwater (*Chlorella* sp. L38) and marine (*Phaeodactylum tricornutum* MASCC-0025) environment. To intensify the biodegradation performance, algae cultivation condition was studied and optimized under different SDM initial concentrations. Meanwhile, the antibiotic removal rate is further intensified *via* increasing the salinity of cultivation medium.

## Materials and Methods

### Microalgae Species

Two microalgae (*Chlorella* sp. L38 and *P. tricornutum* MASCC-0025) were used as target strains, since existing literatures have indicated that *Chlorella* sp. has an excellent capacity on environmental purification. Meanwhile, marine environment is also an important contaminator *via* antibiotics. Therefore, *P. tricornutum* MASCC-0025 is also investigated for antibiotic removal. *Chlorella* sp. L38 and *P. tricornutum* MASCC-025 were obtained from the algae collection of Applied Microalgae Biology Laboratory of Ocean University of China and the Algae Collection of Institute of Oceanography, Chinese Academy of Sciences. SDM is selected as the representative antibiotic for all the experiments, and it was purchased from Aladdin Chemistry Co., Ltd. (Shanghai, China).

### Cultivation Conditions

The cultivation media for *Chlorella* sp. L38 and *P. tricornutum* MASCC-0025 are BG11 and f/2, respectively. Different SDM concentrations (0.5, 1, and 3 mg/L) were added to the cultivation media. To investigate the influence of high salinity on SDM removal performance, different concentration of NaCl solution is added into freshwater microalgae cultivation media. Both SDM and NaCl were added into media after filtration (0.45 μm polytetrafluoroethylene/PTFE) for bacteria removal. The temperature and illumination conditions were 25 ± 1°C and 5,000 Lux under 24 h. Three parallel samples were set in each group to eliminate the interference of other factors.

### Parameter Determination

The algal biomass variation can be evaluated *via* optical density (OD) value and specific growth rate determination, and the detailed determination and calculation procedures of *Chlorella* sp. L38 have been described in our previous work (Song et al., 2020). The specific growth rate (μ) was measured by fitting the dry cell weight (DCW, μg/L) to an exponential function using the equation proposed by Kabra et al. (2014). The relationship between OD_450_ and DCW of *P. tricornutum* (PT) MASCC-0025 was evaluated by the following equation:


(1) $$\mathrm{DCW}\mathrm{of}\mathrm{PT}\left(g/L\right)=0.3109\cdot {\text{OD}}_{450}+0.0007,{\text{R}}^{2}=0.09985$$

In addition, pH of cultivation media was measured every 2 days. Chlorophyll (*a* and *b*) and carotenoids were detected every 4 days in the whole cultivation period. The total chlorophyll content was measured by Kurade et al. (2016). In addition, the superoxide dismutase (SOD), malondialdehyde (MDA), and catalase (CAT) enzyme contents were determined *via* corresponding methods. The potential degradation product of SDM is analyzed *via* LC-MS (e2695 system; Waters Co., Ltd., United States).

### Statistical Analysis

One-way ANOVA was used for statistical analysis. Results were expressed as means ± SEM based on parallel experiments and were considered at 95% CIs.

## Results and Discussion

### Microalgae Growth

**FIGURE 1:**
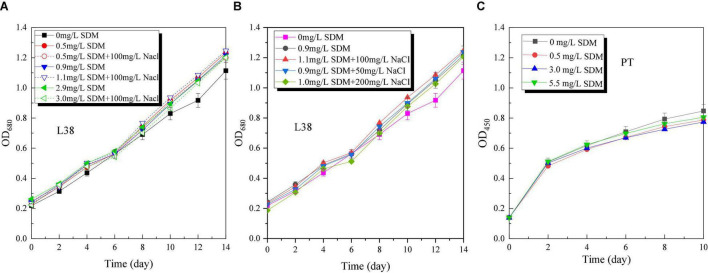
Optical density (OD) variation of *Chlorella* sp. L38 and *Phaeodactylum tricornutum* MASCC-0025 under different sulfadimethoxine (SDM) concentrations.

**FIGURE 2:**
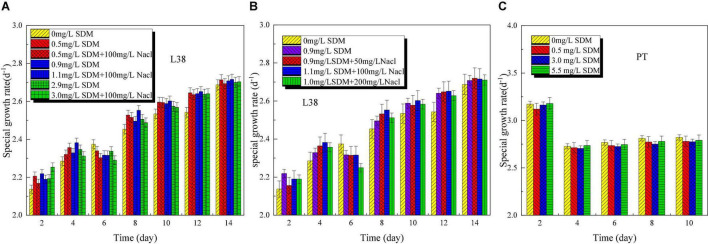
Specific growth rate variation of *Chlorella* sp. L38 and *Phaeodactylum tricornutum* MASCC-0025 under different sulfadimethoxine (SDM) concentrations.

Microalgae growth performance (OD, specific growth rate, and pH) of *Chlorella* sp. L38 and *P. tricornutum* MASCC-0025 under different SDM concentrations is shown in Figure 1. From Figure 1A, it can be seen that when the concentration was lower than 3 mg/L, SDM presented a certain stimulating effect on the growth of *Chlorella* sp. L38. Especially, when the initial concentration of SDM was set at 0.5 mg/L, the growth rate of *Chlorella* sp. L38 was significantly higher than that of the control group (without SDM addition). It could also be observed that SDM had a significant inhibitory effect on the growth of *Chlorella* sp. L38 on the 6th day, and the inhibitory effect increased with SDM concentration. Associated with growth, from the 8th to the 14th days, *Chlorella* sp. L38 gradually adapted the cultivation condition with adding SDM, and the OD value presented an increased trend and specific growth rate also became higher (as shown in Figure 2A). According to Figures 1B, 2B, for different concentration gradients of SDM with and without adding NaCl solution, it can be seen that the growth rate of *Chlorella* sp. 38 increased with the increase of salt concentration. When the addition dosage of NaCl increased to a certain extent, the growth promotion effect on *Chlorella* sp. L38 would decrease with the continuous increase of salt concentration. It indicated that a certain amount of NaCl can promote *Chlorella* sp. L38 growth, and it could be also seen that the facilitation effect of the salt on *Chlorella* sp. L38 growth would vary with initial SDM concentration.

For *P. tricornutum* MASCC-0025, the OD and specific growth rate variation are shown in Figures 1C, 2C. It could be found that the presence of SDM had a slight inhibitory effect on the growth of algae, and the inhibitory effect was the strongest at the SDM concentration of 3 mg/L. However, when the concentration of SDM increased to 5.5 mg/L, the inhibitory effect on microalgae slowed down. It could be observed that for the different microalgae species, the SDM effect was also different. Therefore, to select suitable microalgae strain for antibiotics degradation, more efforts should be paid on the investigation of the metabolism mechanism and genetic modification of microalgae.

**FIGURE 3:**
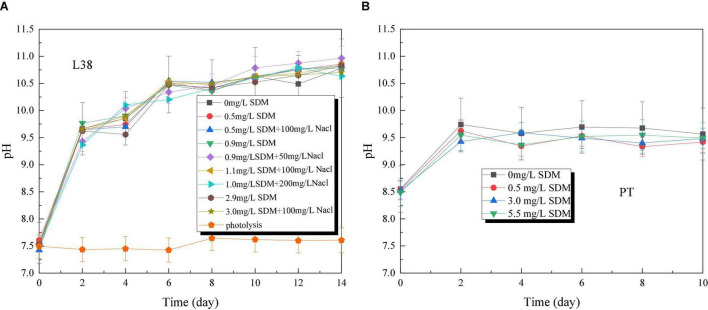
pH variation of *Chlorella* sp. L38 and *Phaeodactylum tricornutum* MASCC-0025 under different sulfadimethoxine (SDM) concentrations.

The pH of *Chlorella* sp. L38 increased rapidly from about 7.5 to 11 in the initial 2 days of cultivation, and then varied slowly from 4 to 14 days (maintained at 10.5–11.0), as shown in Figure 3A. It also indicated that *Chlorella* sp. L38 has a good adaptive capacity to SDM. For *P. tricornutum* MASCC-0025, the pH value of all the groups varied around 9.5, and the control group was slightly higher than that of the other groups with adding SDM, as illustrated in Figure 3B. It might be due to the inhibitory effect of antibiotics on the growth of *P. tricornutum* MASCC-0025, leading to the growth state of the experimental group that was slightly slower than that of the control group. Overall, the pH variation trend was consistent with algae biomass accumulation.

### Antioxidant Enzyme Variation

Usually, reactive oxygen species (ROS), namely superoxide anions (O^2–^) and hydrogen peroxide (H_2_O_2_), are important signaling molecules that control cell metabolism. They play a key role in the development, growth, differentiation, and proliferation of multicellular organisms, which are usually stimulated by organic pollutants (Vaahtera et al., 2014; Zandalinas and Mittler, 2018). On the other hand, excessive ROS has a risk to damage the microbial membrane system and ultimately impedes growth. To protect against and eliminate the toxicity caused by ROS, microalgae could secrete various antioxidant enzymes through antioxidant defense mechanisms, such as SOD and CAT (Xiong et al., 2017a). For example, as an important antioxidant enzyme, SOD can transfer O^2–^ to H_2_O_2_ and O_2_ to avoid its accumulation. When algae are stressed by low concentrations of exogenous pollutants, it can avoid or reduce oxidative damage by increasing the activity of these antioxidant enzymes in microalgae cells. However, when the tolerable threshold is exceeded, serious oxidative damage to cell structure and even death may result. As the main product of lipid peroxidation, MDA content can represent the damage degree of cell membrane.

**FIGURE 4:**
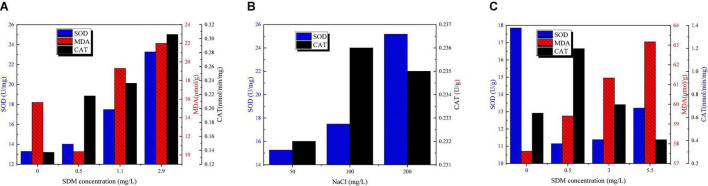
Superoxide dismutase (SOD), malondialdehyde (MDA), and catalase (CAT) content variation of *Chlorella* sp. L38 and *Phaeodactylum tricornutum* MASCC-0025 under different sulfadimethoxine (SDM) concentrations.

Figure 4 shows SOD, MDA, and CAT concentration variation of *Chlorella* sp. L38 and *P. tricornutum* MASCC-0025 under different SDM concentrations. According to Figure 4A, due to the presence of SDM, O^2–^ increased rapidly associated with the growth of *Chlorella* sp. L38, resulting in the corresponding increase of SOD in the algal cell. The amount of O^2–^ was positively correlated to the increase of SDM concentration. In Figure 4B, it could be also seen that the addition of NaCl could promote the generation of SOD and CAT enzymes in *Chlorella* sp. L38. Thus, increasing salinity of growth condition facilitated to alleviate the toxicity of SDM to *Chlorella* sp. L38. It should be noted that when the NaCl concentration increased to 200 mg/L, the CAT enzyme content would be decreased and lower than that of 100 mg/L NaCl. It might be because the high salt concentration adversely affected the transport of substances. Therefore, under the action of SOD enzyme, O^2–^ would be transferred into H_2_O_2_ but not decomposed into H_2_O and O_2_ in time. In Figure 4A, when the concentration of SDM was set at 0.5 mg/L, the SOD and CAT enzymatic reactions of algal cells were intensified and the stress resistance was enhanced. When the concentration of SDM varied from 1 to 3 mg/L, with the increase of the concentration of antibiotics, the equilibrium reaction was more destructive, and thus the content of MDA increased. However, in the group of adding NaCl, it was observed that salt presented a certain maintenance effect on the balance of the reaction, and the content of MDA in algal cells was much lower than that in the group without adding NaCl.

For *P. tricornutum* MASCC-0025, as shown in Figure 4C, it can be seen that with the increase of the concentration of antibiotics, the content of MDA and SOD also increased, indicating that the degree of algal cell damage increased. However, when SDM concentration was set at 1 mg/L, it could be seen that SOD enzyme concentration was significantly lower than that in the control group, while CAT enzyme concentration was the highest. That might be since at the initial stage of *P. tricornutum* MASCC-0025 cultivation, part of O^2–^ has been converted into H_2_O_2_. Thus, due to the reduction of O^2–^, the content of SOD decreased. Meanwhile, the increased H_2_O_2_ content would stimulates the production of CAT enzymes in algae cells to continuously convert H_2_O_2_ into H_2_O and O_2_.

### Chlorophyll Variation

**FIGURE 5:**
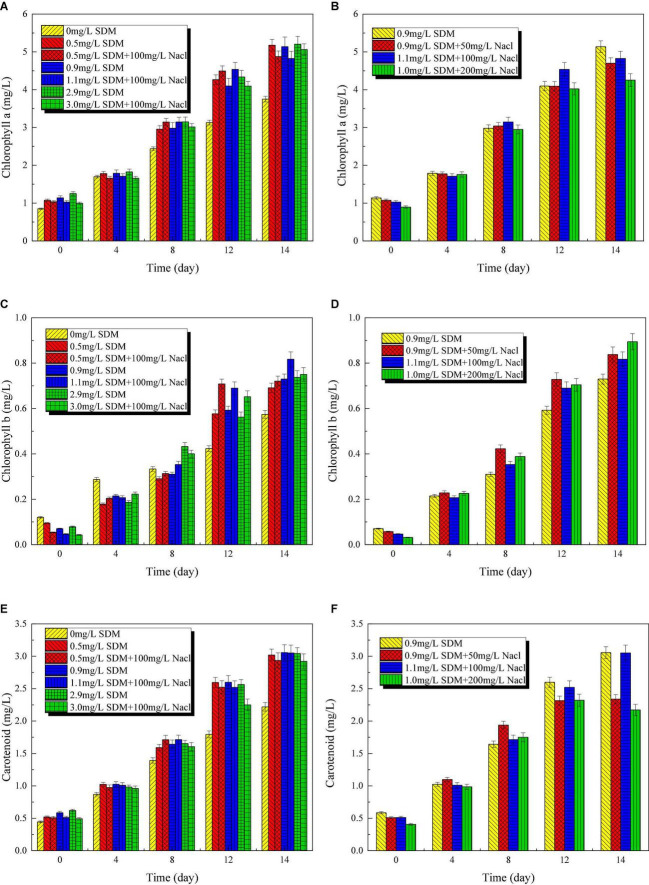
Chlorophyll *a* **(A,B)**, *b* **(C,D)**, and carotenoid **(E,F)** content variation of *Chlorella* sp. L38 and *Phaeodactylum tricornutum* MASCC-0025 under different sulfadimethoxine (SDM) concentrations.

The photosynthetic processes of microalgae include the photosystem I (PSI) and the photosystem II (PSII) (Mirkovic et al., 2017). For PSI, the light collection efficiency of photosynthesis is related to chlorophyll *a*. The content of chlorophyll *a* is important for assessing the adaptability of microalgae to environmental stresses such as salinity. Chlorophyll *a*, *b* and carotenoid content variation of *Chlorella* sp. L38 under different SDM concentrations is shown in Figure 5. It should be pointed out that the chlorophyll content of *P. tricornutum* MASCC-0025 is too low to be detected. From Figures 5A,B, it can be seen that SDM had a potential to promote the synthesis of chlorophyll *a* at 8–14 days. By contrast, the addition of SDM would inhibit the synthesis of chlorophyll *b* within 0–4 days, and then promoted the synthesis of chlorophyll *b* after the later adaptation. Under the same SDM concentration, with the increase of NaCl concentration, the synthesis of chlorophyll *a* in *Chlorella* sp. L38 cells was inhibited. Compared with chlorophyll *a*, chlorophyll *b* showed no obvious inhibition or promotion at the initial stage of culture, as shown in Figures 5B,C. With the increase of time, the synthesis of chlorophyll *b* was promoted after 8 days. Meanwhile, from 12 days of cultivation, the presence of SDM significantly promoted the synthesis of carotenoids, as illustrated in Figures 5E,F. Within 0–8 days, the presence of NaCl had a slight stimulating effect on the synthesis of carotenoids. However, with the increase of salt concentration, the synthesis of carotenoids decreased. At 12–14 days, the presence of salt turned to inhibit the synthesis of carotenoids. The aforementioned results indicated that *Chlorella* sp. L38 could adapt to the cultivation environment containing SDM and NaCl. It should be noted that the synthesis of chlorophyll and carotenoids in microalgae might be also inhibited if the NaCl concentration was too high. The reason might be that except Na^+^ absorbed by microalgae, Cl^–^ could not be utilized, and which had a certain toxic effect on microalgae, thus inhibiting the synthesis of chlorophyll and carotenoids.

### Sulfadimethoxine Removal Performance

**FIGURE 6:**
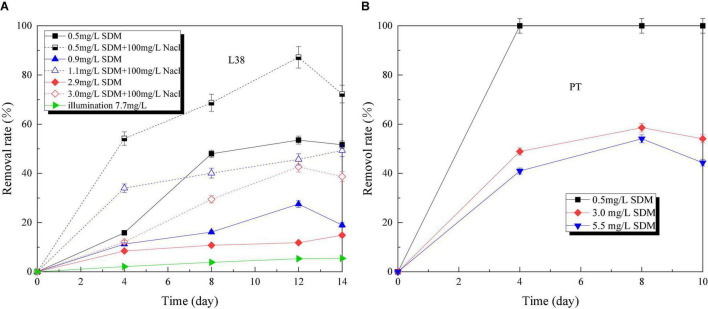
Sulfadimethoxine (SDM) removal rate variation of *Chlorella* sp. L38 and *Phaeodactylum tricornutum* MASCC-0025 under different initial concentrations.

Figure 6 presents SDM removal rate of *Chlorella* sp. L38 and *P. tricornutum* MASCC-0025 under different initial concentrations. The experiment proved that SDM almost could not be degraded under the condition of light alone. Also, the maximum degradation rate reached about 88% after *Chlorella* sp. L38 were introduced, as shown in Figure 6A. Yu et al. (2011) proposed that SDM could be removed by 52% through biological action in the immobilized cell system. By comparing different NaCl concentration gradients under the same SDM initial dosage, it could be seen that certain salt concentrations could stimulate *Chlorella* sp. L38 to degrade SDM. On the 14th day of cultivation, the degradation times achieved up to 3.3 times compared with the group without adding NaCl. For example, when the initial SDM concentration was set at 1 mg/L, the degradation rate of microalgae with adding 100 mg/L NaCl reached 50% after 14 days of cultivation. This was because under salt-treated conditions, microalgae could regulate their growth at low NaCl concentrations by altering their biochemical properties, such as enzyme systems and extracellular polymers (EPS). Xiong et al. (2017b) also indicated that microalgae could present an increased photosynthetic activity (enhanced CO_2_ assimilation by transferring carbon and energy resource into algal biomass) and adaptive mechanism under salt stress.

Compared with *Chlorella* sp. L38, *P. tricornutum* MASCC-0025 showed a good SDM removal performance. Under the SDM concentration at 3.0 mg/L, the SDM removal rates of *Chlorella* sp. L38 (adding NaCl) and *P. tricornutum* MASCC-0025 were 29.45 and 58.59% (1.98 times higher than *Chlorella* sp. L38), respectively. It could be seen that marine algae might have a superiority to be a potential option to remove SDM.

### Metabolic Product Analysis

To further investigate biodegradation pathway of SDM, its microalgal metabolic products were also investigated. Kiki et al. (2020) proposed that microalgal biodegradation could reduce the toxicity of antibiotics and then transform them into less toxic intermediates. Xiong et al. (2019) found that the major bioconversion pathways of SMZ were hydrolysis, methylation, dechlorination, and hydroxylation, and the main pathways of SMX degradation were nitrosation, deamination, methylation, and hydroxylation. As another typical member of sulfonamide antibiotics, Sun et al. (2018) proposed that due to the similarity of SDM and SMX structures (side chain groups of amino substituted benzene ring), their degradation process and degree might be similar. According to the identification of metabolites *via* LC-MS, metabolic pathway of SDM *via Chlorella* sp. L38 was mainly through deamination reaction to generate hydroxyl and amino compounds.

## Conclusion

The biodegradation fate of SDM *via Chlorella* sp. L38 and *P. tricornutum* MASCC-0025 was investigated and the biodegradation performance was optimized *via* cultivation condition variation. The experimental results indicated that *Chlorella* sp. L38 could better adapt SDM addition *via* antioxidant enzyme secretion. In addition, moderate salinity condition could stimulate SOD (25.18 U/mg) and CAT (0.24 nmol/min/mg) enzyme generation. By contrast, *P. tricornutum* MASCC-0025 has the advantage of high salinity to stimulate SDM degradation rate (up to 100%). It could be observed that microalgae have the potential for antibiotics removal from freshwater and marine environment under the optimized species and cultivation conditions.

## Data Availability Statement

The raw data supporting the conclusions of this article will be made available by the authors, without undue reservation.

## Author Contributions

BL: data curation, original draft preparation, revising and software. DW: writing, reviewing, data curation, original draft preparation, and software. YL: original draft preparation, reviewing and editing. YS: reviewing and editing. CW and JS: data curation. CS: conceptualization, methodology, writing, reviewing, editing and supervision. All authors contributed to the article and approved the submitted version.

## Conflict of Interest

The authors declare that the research was conducted in the absence of any commercial or financial relationships that could be construed as a potential conflict of interest.

## Publisher’s Note

All claims expressed in this article are solely those of the authors and do not necessarily represent those of their affiliated organizations, or those of the publisher, the editors and the reviewers. Any product that may be evaluated in this article, or claim that may be made by its manufacturer, is not guaranteed or endorsed by the publisher.
