# Supplementary material for: Metabolic Mechanism of Sulfadimethoxine Biodegradation by Chlorella sp. L38 and Phaeodactylum tricornutum MASCC-0025
Source: Front Microbiol. 2022 Mar 18;13:840562. doi: 10.3389/fmicb.2022.840562 (PMC8971708; doi:10.3389/fmicb.2022.840562)
Supplement: Supplementary file 1 [file Data_Sheet_1.PDF]

Supplementary Material:

Biodegradation and metabolic mechanism of sulfadimethoxine via *Chlorella sp.* L38 and *Phaeodactylum tricornutum* MASCC-0025

Bing Li [a], Di Wu [a], Yan Li [a], Yan Shi [a], Chenlin Wang [b], Jiasi Sun [b], Chunfeng Song [b, *]

[a] *The Institute of Agriculture Resources and Environmental Sciences, Tianjin Academy of Agricultural Sciences. 26 Hangtian Road, Nankai District, Tianjin 300-192, China.*

[b] *Tianjin Key Laboratory of Indoor Air Environmental Quality Control, School of Environmental Science and Engineering, Tianjin University, 92 Weijin Road, Nankai District, Tianjin, P.R. China*

* Corresponding author. Tel: +86-022-8740-1255.

E-mail: chunfeng.song@tju.edu.cn

Table S1 Metabolites identification of SDM by UPLC–MS after 14 days of cultivation of *Chlorella* sp. L38.

No.	t_R (min)	Mw	MS (m/z)	Mass spectra
SDM	8.5	310	349 [M+K]$^+$	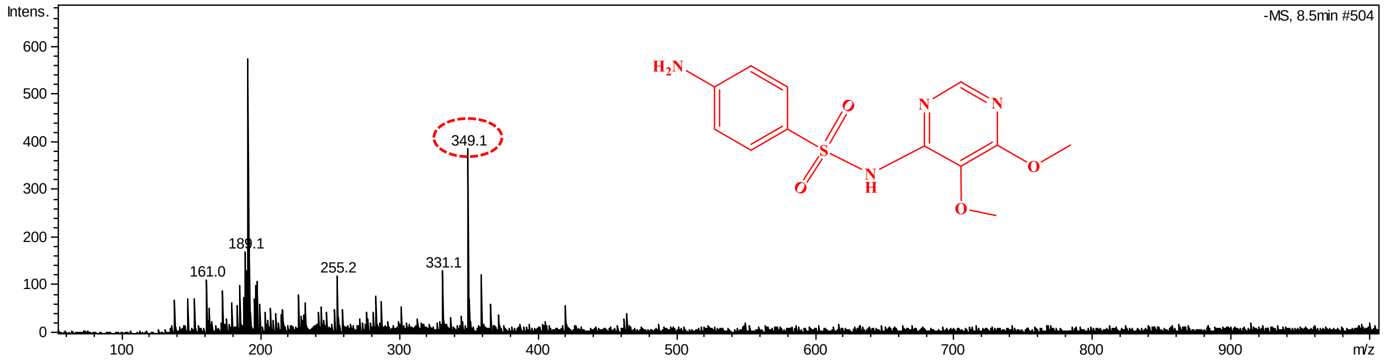
			309 [M-H]$^-$	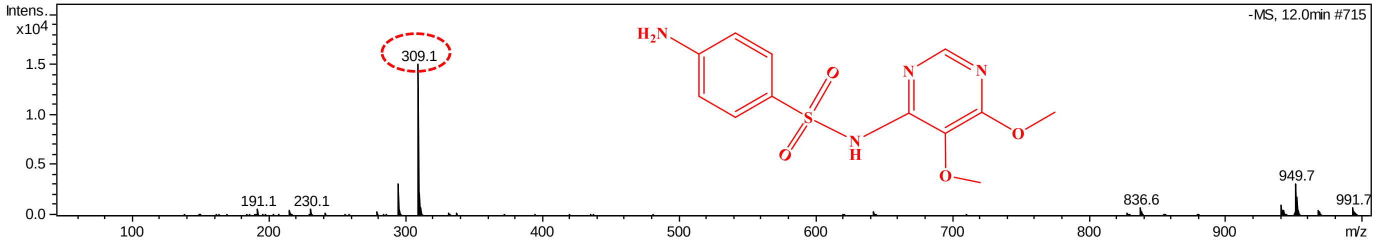

I	10.8	173	191<br>$[M+H_2O]^+$	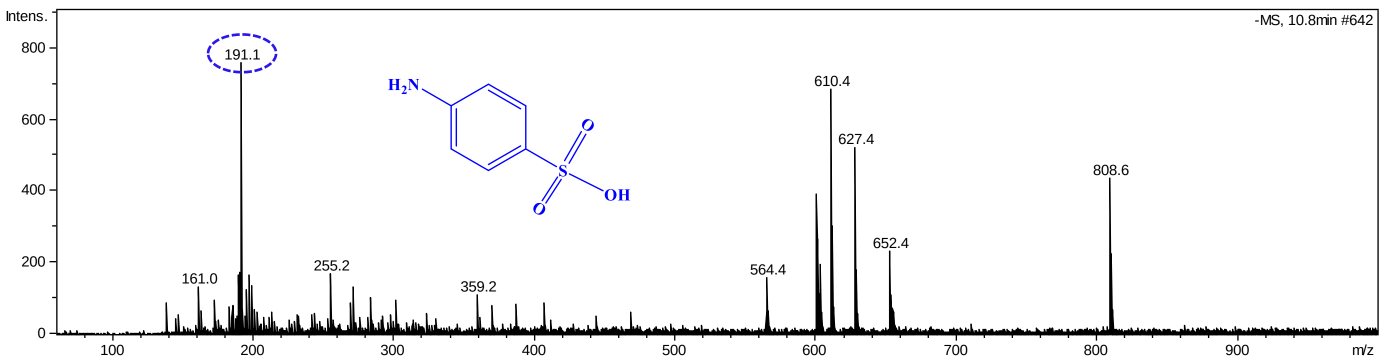
II	1.6	172	211<br>$[M+K]^+$	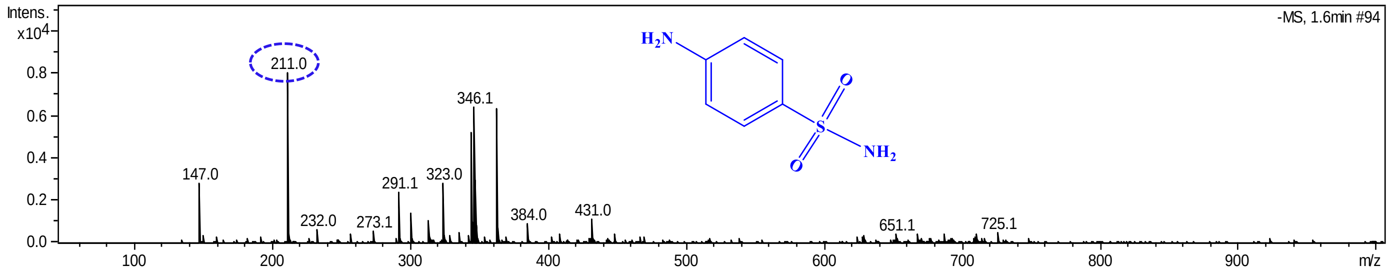
